# Matched embryo–endometrium RNA-seq reveals coordinated but asymmetric transcriptomic reprogramming at the onset of early equine pregnancy

**DOI:** 10.3389/fcell.2026.1840498

**Published:** 2026-06-18

**Authors:** Eva Da Silva Álvarez, José M. Ortiz-Rodríguez, Francisco E. Martín-Cano, Alberto Álvarez-Barrientos, Laura Becerro-Rey, María C. Gil, Eloy Redondo, Javier Masot, Inés M. Aparicio, Fernando J. Peña, Cristina Ortega-Ferrusola

**Affiliations:** 1 Laboratory of Equine Reproduction and Equine Spermatology, Veterinary Teaching Hospital, University of Extremadura, Cáceres, Spain; 2 Department of Veterinary Clinical Sciences, Alma Mater Studiorum, University of Bologna, Bologna, Italy; 3 Service of Techniques Applied to Biosciences, University of Extremadura, Badajoz, Spain

**Keywords:** conceptus, early pregnancy, endometrium, horse, RNA-seq, transcriptomics

## Abstract

**Background:**

Maternal recognition of pregnancy (MRP) in the mare requires coordinated adaptations of both the conceptus and the endometrium during the peri-recognition interval; however, integrated transcriptomic analyses of both compartments within the same biological window remain limited. Here, we analyzed conceptus RNA-seq data across embryonic age (Days 8 and 12 post ovulation) and endometrial RNA-seq data across pregnancy status (pregnant versus non-pregnant mares sampled between Days 8 and 12 post ovulation). In pregnant mares, conceptus recovery and endometrial biopsy collection were performed within the same gestation, allowing biological integration of embryo and maternal transcriptomes while preserving tissue-specific statistical contrasts.

**Results:**

RNA-seq analysis followed by TMM normalization and voom–limma modeling identified differential expression in both compartments. Differential expression was defined by an adjusted P-value (FDR threshold <0.001). The B-statistic (log-odds of differential expression) was used to prioritize high-confidence candidates for interpretation and downstream modeling, considering genes with a Bayesian posterior probability >1. Gene-level interpretation was restricted to transcripts with a Bayesian posterior probability of differential expression (B > 1). In the conceptus, high-confidence upregulated genes from day 8 to 12 included steroidogenic enzymes (*CYP19A1, CYP17A1*), extracellular matrix components (*COL4A1, COL4A2, COL4A5, SPON1, DSG2*), protease regulators (*SERPINE1, TMPRSS2, LGMN*), and selective transporters (*AQP5, SLC2A5, ATP1B3*). In the endometrium, downregulated genes in pregnant mares included immediate-early transcriptional regulators (*EGR1, FOS, DUSP1*) and oxidative stress–associated genes (*GSTA4*), while upregulated genes in pregnant mares included signaling and regulatory components (*HRAS, PUM3, U2AF1L4, COPS6*), complement regulator *C4BPA*, and mitochondrial sulfide metabolism gene *SQOR*. Functional enrichment analysis supported coordinated extracellular matrix organization and signaling modulation without enrichment of inflammatory pathways.

**Conclusion:**

Matched transcriptomic profiling reveals coordinated but compartment-specific gene regulation at the onset of early equine pregnancy. The conceptus exhibits endocrine and interface specialization, whereas the endometrium demonstrates attenuation of immediate-early transcriptional programs and selective signaling recalibration. These data define a high-confidence systems-level framework for early embryo–maternal communication that precedes the classical maternal recognition phase and is consistent with early embryo–maternal communication associated with the transition toward maternal recognition.

## Introduction

Uterine function during the period of high mobility of the equine embryo is tightly orchestrated by ovarian steroid hormones, particularly estrogen and progesterone, which regulate endometrial receptivity and early conceptus support (Wilsher and Allen, 2009; Gibson et al., 2018; Pinto, 2020). However, maintenance of early pregnancy in the mare ultimately depends on suppression of endometrial prostaglandin F_2_α (PGF_2_α) secretion during the maternal recognition of pregnancy (MRP) window, generally considered to occur between Days 10 and 16 post-ovulation. Although the molecular identity of the equine MRP signal remains unresolved, functional studies support conceptus-dependent modulation of prostaglandin pathways and oxytocin responsiveness within this interval. This indicates that embryo–maternal communication must begin prior to the classical luteolytic phase and may involve coordinated local regulatory processes rather than a single linear endocrine mediator, as recently emphasized in comprehensive analyses of equine MRP (Swegen, 2021).

Successful fixation and subsequent implantation require precise synchrony between a developmentally competent embryo and a receptive endometrium. In humans, this synchrony is defined by a narrow “window of implantation,” occurring approximately between Days 7–9 post-ovulation, during which inadequate endometrial receptivity accounts for up to two-thirds of implantation failures (Das and Holzer, 2012). Profound and coordinated changes in endometrial gene expression characterize this window, with deregulation leading to implantation failure and infertility (Vasquez et al., 2018; Sehring et al., 2022; Juarez-Barber et al., 2024; Xu et al., 2025). In contrast, the horse does not exhibit such a sharply restricted implantation window, as evidenced by the broad range of acceptable embryo–recipient asynchrony in equine embryo transfer programs (Wilsher et al., 2010; Jacob et al., 2012). Nevertheless, in vitro-produced equine embryos display a markedly narrower window of synchrony for successful pregnancy compared with their in vivo-derived counterparts (Cuervo-Arango et al., 2019a; Cuervo-Arango et al., 2019b). One plausible explanation is the absence of early embryo–endometrium cross-talk during *in vitro* development, supporting the hypothesis that molecular communication between the conceptus and the uterus begins at very early stages and contributes to developmental competence.

In several species, including humans, mice, cattle, and pigs, the viable embryo secretes the Preimplantation Factor (PIF), a 15-amino acid peptide detectable as early as the two-cell stage (Roussev et al., 1996; Moindjie et al., 2016; Shainer et al., 2016; Wonfor et al., 2017; Santos et al., 2021). Although PIF has not been identified in the equine embryo, exposure of equine endometrial explants to synthetic PIF induces measurable biological responses, particularly in the modulation of inflammatory pathways (Nash et al., 2018). Immune regulation represents a critical component of early pregnancy establishment, as the maternal immune system must tolerate the semi-allogeneic embryo while preserving antimicrobial defense (Paidas et al., 2010; Walker et al., 2010). In the mare, where insemination induces a transient but robust inflammatory response (Da Silva et al., 2024; Da Silva-Alvarez et al., 2026), controlled immune modulation during the pre-implantation period may be particularly relevant.

Transcriptomic studies in the mare during the first days of gestation indicate that embryo–maternal communication is initially subtle but biologically specific at the cellular level. While no global changes in endometrial gene expression are detected at Day 8 of pregnancy, differential transcriptomic profiles associated with the presence of the conceptus emerge between Days 10 and 12 post-ovulation (Merkl et al., 2010; Pinto, 2020). These changes are primarily localized to the luminal epithelium, where genes involved in estrogen signaling, nutrient transport, and uterine metabolism are selectively regulated, including repression of the estrogen receptor *ESR1* and induction of amino acid transporters such as *SLC36A2,* consistent with early metabolic adaptation of the endometrium to conceptus demands (Klein et al., 2010; Klohonatz et al., 2019a). The luminal epithelium represents the principal responsive compartment during the initial phase of maternal recognition of pregnancy, exhibiting differential regulation of genes associated with prostaglandin signaling and transport, growth factors (*FGF9, IGFBP2*), vascular remodeling, and epithelial–conceptus communication, in close association with conceptus size and developmental stage (Rudolf Vegas et al., 2021).

Early transcriptomic investigations in the horse have provided evidence for embryo-associated endometrial responses during the pre-implantation period but remain limited by the absence of direct matched embryo–endometrium comparisons within the same gestation. Analyses based on asynchronous embryo transfer models or endometrium-only profiling have revealed embryo-dependent regulation of immune, inflammatory, and prostaglandin-related pathways, yet without simultaneous transcriptomic assessment of the corresponding conceptus (Klohonatz et al., 2019b; Gibson et al., 2020). Collectively, these observations support the hypothesis that the equine embryo influences endometrial function prior to fixation and before the classical prostaglandin suppression phase of maternal recognition. However, the extent to which embryonic developmental reprogramming and endometrial adaptation are temporally coordinated within the same gestation remains incompletely defined. Next-generation sequencing (NGS) therefore, provides a powerful strategy to characterize these early bilateral transcriptional dynamics. Based on previous evidence that embryo-dependent endometrial responses emerge during the pre-implantation period in the mare, we aimed to determine whether conceptus developmental progression and the maternal endometrial response show coordinated transcriptomic signatures within the same early gestational window. Specifically, we compared conceptus transcriptomes between Days 8 and 12 post ovulation and, separately, endometrial transcriptomes from pregnant and non-pregnant mares sampled between Days 8 and 12 post ovulation, followed by integrative interpretation of the two tissue-specific datasets. We hypothesized that these analyses would reveal coordinated, but compartment-specific, molecular programs associated with the onset of early equine pregnancy.

## Materials and methods

### Embryo and biopsy collection and experimental design

Animals were maintained according to European regulations, and all experimental procedures were approved by the Ethical Committee of the University of Extremadura (Ref#PID 2021–122351OB-I00 Cáceres, Spain). To synchronize estrous cycles, mares received a prostaglandin analogue and were monitored daily by transrectal ultrasonography for follicular development, uterine edema, and cervical tone. When a dominant follicle (≥35 mm × 35 mm) was detected in the absence of luteal tissue, mares were treated with 2,500 IU hCG. Ovulation was confirmed by ultrasonography, and mares were inseminated at ovulation with semen from the same fertile stallion in order to minimize paternal variability. Pregnancy status was assessed by power Doppler evaluation of uterine vascularization (Nieto-Olmedo et al., 2020). In pregnant mares, conceptus recovery was performed by uterine lavage, immediately followed by a single endometrial biopsy collection from the uterine body using Kessler biopsy forceps; biopsies were taken from the same uterine location in all the mares after uterine lavage. Each biopsy measured approximately 2 cm in length and 0.5 cm in thickness and consisted of uterine mucosa, including luminal and glandular compartments. All samples were snap-frozen and stored at −80 °C until RNA extraction.

The study was designed as a combined matched-sampling and parallel-comparison framework. In pregnant mares, conceptus and endometrial samples were obtained from the same gestation and subsequently integrated at the interpretative level. For differential expression analyses, however, each tissue was analyzed according to its own biological contrast: conceptus transcriptomes were compared across developmental stage (Day 8 versus Day 12 post ovulation), whereas endometrial transcriptomes were compared across pregnancy status (pregnant versus non-pregnant mares sampled between Days 8 and 12 post ovulation) Initially, 10 libraries were obtained from 8-day embryos and 16 from 12-day embryos. For the endometrial biopsies, 6 non-pregnant and 4 pregnant at day 8, and 7 non-pregnant and 5 pregnant libraries were created for day 12. This design allowed us to examine embryonic developmental progression and the maternal pregnancy-associated response within the same biological window, while avoiding direct statistical equivalence between the two tissue compartments.

### RNA extraction

We extracted RNA from endometrial biopsies and embryos using the PicoPure™ RNA Isolation Kit (ThermoFisher) total RNA procedure. The extracted RNA was consistently highly concentrated and of high quality, as indicated by the Agilent Bioanalyzer using the Agilent RNA 6000 Nano Kit. The RIN was ≥8, with an average concentration of 56.6 ± 8.2 ng/μL.

### Library preparation

All samples were processed identically; mRNA enrichment was performed from total RNA using the MultiMACS mRNA Isolation Kit (Miltenyi Biotec). RNA integrity of the enriched mRNA fraction was evaluated on an Agilent 2100 Bioanalyzer using the Agilent RNA 6000 Pico Kit, showing the expected size distribution and low rRNA contamination, consistent with high-quality poly(A)+ RNA. The average concentration of enriched mRNA was 2.2 ± 1.3 ng/μL. Sequencing libraries were prepared for the Ion S5 XL System using the Ion Total RNA-Seq Kit v2 (Thermo Fisher Scientific; Cat. No. 4479789) according to the manufacturer’s instructions. Poly(A)+ RNA was used as input. RNA fragmentation with RNase III was performed at 37 °C for 3 min as recommended by the kit protocol. Fragmented RNA was purified using the Magnetic Bead Cleanup Module (Thermo Fisher Scientific; Cat. No. 4475486) and eluted in 13 µL nuclease-free water. Adapter ligation was carried out using the Ion RNA-Seq Primer Set v2 (Thermo Fisher Scientific; Cat. No. 4479789). For each sample, 3 µL of purified fragmented RNA were combined with 2 µL Ion Adapter Mix v2 and 3 µL Hybridization Solution, and incubated in a thermal cycler at 65 °C for 10 min followed by 30 °C for 5 min. Subsequently, 10 µL 2× Ligation Buffer and 2 µL Ligation Enzyme Mix were added, and the reaction was incubated at 30 °C for 1 h. Reverse transcription (RT) was performed using the kit reagents. An RT master mix (nuclease-free water, 10× RT buffer, dNTP mix, and Ion RT Primer v2) was added to each ligation reaction and incubated at 70 °C for 10 min, followed by stepwise cooling per the manufacturer’s protocol. SuperScript Enzyme Mix was then added, and RT was carried out at 42 °C for 30 min.

The resulting cDNA was purified using the Magnetic Bead Cleanup Module and eluted in 12 µL nuclease-free water. For library amplification, 6 µL of cDNA were mixed with 45 µL Platinum PCR SuperMix, 1 µL Ion Xpress 3′ Barcode Primer, and 1 µL Ion Xpress RNA Barcode (Thermo Fisher Scientific; Cat. No. 4475485). PCR amplification was performed in a thermal cycler using the cycling conditions recommended in the Ion Total RNA-Seq Kit v2 user guide (hold 2 min 94 °C; Cycle 2× [94 °C 30 s; 50 °C 30 s; 68 °C 30 s]; cycle 16× [94 °C 30 s; 62 °C 30 s; 68 °C 30 s]; Hold 5 min 68 °C). The amplified cDNA was purified using the Magnetic Bead Cleanup Module and analyzed by capillary electrophoresis on an Agilent 2100 Bioanalyzer using the High Sensitivity DNA Kit (Agilent Technologies; Cat. No. 5067-4626).

### Sequencing on ion S5 Xl platform

Sequencing libraries were diluted to equimolar concentrations of 50 pM and pooled prior to template preparation. Library pools were loaded onto the Ion Chef™ System using the Ion 540™ Kit-Chef (Invitrogen; Cat. No. A30011), which contains pre-packaged template reagent cartridges, following the manufacturer’s instructions. Prepared templates were subsequently loaded onto Ion 540™ chips (Invitrogen; Cat. No. A27765). Sequencing was performed on the Ion S5™ XL System (Thermo Fisher Scientific) using the Ion S5 Sequencing Solutions and reagents provided in the Ion 540™ Kit-Chef. The median read length was 122.4 ± 20.3 nucleotides, and each library generated approximately (1–2) × 10^7^ reads. Sequencing quality was assessed using the AQ20 metric, defined as the longest read length with an error rate ≤1%. Across all libraries, 88.3% ± 0.5% of reads achieved Q20 or higher.). The processed data supporting the conclusions of this article are included in the article and its Supplementary Material. The raw BAM files are archived in the institutional repository of the University of Extremadura under controlled access. Access to these files can be provided by the corresponding author upon reasonable request through a secure link and password-protected access.

### Bioinformatic Analysis

#### Computational workflow for RNA-seq quantification, normalization, and differential expression analysis

Pre-quality control (QC); the dataset comprised 26 embryo libraries and 22 endometrial biopsy libraries derived from six mares, with some individuals contributing samples at multiple time points. To prevent carry-over inflammation or altered endometrial response due to repeated sampling, mares were subjected to a strict washout period of at least one complete estrus cycle (4 weeks) between procedures. Furthermore, in our bioinformatics pipeline, the variable “mare identity” was included as a blocking factor in the design matrix of the limma-voom model. Because some mares contributed samples at more than one time point, the data had a repeated-measured structure. To account for this repeated-measures structure and avoid inflated significance due to within-mare non-independence, mare identity was included as a blocking factor, and within-mare correlation was estimated using limma’s duplicate correlation framework for downstream differential expression analyses. Samples failing predefined QC criteria (e.g., insufficient library size, abnormal expression distributions, and outliers identified by MDS/PCA; see Supplementary Files) were excluded before final analyses; final sample counts per tissue and time point are reported in the Supplementary Tables. RNA-seq data were processed using a standardized and reproducible computational pipeline. Raw sequencing reads (FASTQ files) were initially quality-checked using FastQC, and adapter sequences and low-quality bases were removed prior to quantification to mitigate platform-specific sequencing artifacts. Transcript-level quantification was performed with Salmon v1.8.0 in selective-alignment mode using a decoy-aware index constructed from the EquCab3.0 genome and the corresponding Ensembl cDNA reference (Ensembl Release 108). Although Ion Torrent sequencing is known to exhibit homopolymer-associated indel errors, the use of selective alignment combined with downstream gene-level summarization minimizes the impact of such errors on expression estimates.

Transcript abundance estimates were summarized to gene-level counts and imported into R v4.2.2 for downstream statistical analysis. Genes with very low expression across samples were filtered out prior to normalization. Library sizes were normalized using the trimmed mean of M-values (TMM) method, and normalized expression values were transformed using voom to model the mean–variance relationship and to generate observation-level precision weights. Differential expression analysis was conducted at the gene level to ensure robustness given the read-length distribution and sequencing characteristics of the Ion Torrent platform.

Differential expression testing was performed using limma’s linear modeling framework. Linear models were fitted with lmFit, incorporating the mare blocking structure and the consensus within-mare correlation estimated by duplicate Correlation, followed by empirical Bayes moderation using eBayes to improve power and stability, particularly in designs with limited sample size and/or multiple factors. Statistical significance was assessed using the Benjamini–Hochberg false discovery rate (FDR) correction, differential expression was defined by an adjusted P-value (FDR threshold <0.001). The B-statistic (log-odds of differential expression) was used to prioritize high-confidence candidates for interpretation and downstream modeling, considering genes with a Bayesian posterior probability >1.

Quality control (QC) procedures included inspection of library sizes, expression distributions, and sample relationships using multidimensional scaling (MDS) and principal component analysis (PCA). All excluded samples, filtering thresholds, and QC metrics are documented in the Supplementary Methods and associated outputs.

The necessity of independent RT-qPCR validation of RNA-seq results has been increasingly questioned, as modern RNA-seq workflows combined with robust statistical frameworks provide accurate and reproducible quantification of gene expression. In particular, empirical Bayes approaches such as those implemented in limma yield explicit probabilistic estimates of differential expression, enhancing robustness even in complex experimental designs. In this context, additional RT-qPCR validation is not considered mandatory when stringent statistical criteria are applied to define differentially expressed genes (Ritchie et al., 2015; Coenye, 2021).

#### Gene Ontology, pathway, and network analysis

Differentially expressed transcripts were aggregated to gene-level identifiers, and all downstream analyses were performed at the gene level. Because equine functional annotations are comparatively limited, differentially expressed equine genes were mapped to their human orthologs using g: Profiler’s orthology conversion tool (https://biit.cs.ut.ee/gprofiler/orth) (Raudvere et al., 2019; Reimand et al., 2019). Gene Ontology enrichment, pathway analysis, and network-level clustering were subsequently performed using Metascape (http://metascape.org). (Zhou et al., 2019) which integrates multiple annotation resources and provides consolidated functional interpretation.

## Results

Transcript-level quantifications generated by Salmon were summarized to gene-level counts, and all downstream analyses were performed at the gene level. Differential expression was evaluated using limma–voom with TMM normalization and empirical Bayes moderation (Materials and Methods). Throughout the Results, we prioritize genes with strong statistical support (B-statistic >1) when highlighting candidates and constructing the integrative model; complete differential expression outputs are provided in Supplementary Tables.

### Differential expression overview of endometrial genes in pregnant *versus* non-pregnant mares

#### Upregulated endometrial genes

Nineteen endometrial genes met the high-confidence criterion (B > 1) for upregulation in pregnant versus non-pregnant mares (Supplementary Table S1), with adjusted P values spanning 1.99 × 10^−5^ to 3.19 × 10^−2^ and log_2_ fold changes ranging from 2.61 to 6.61. The most strongly supported increases included regulators of RNA/protein processing (*PUM3, U2AF1L4, COPS6*), intracellular signaling (*HRAS*), complement modulation (*C4BPA*), and mitochondrial sulfide/redox metabolism (*SQOR),* together with genes linked to vesicle trafficking and membrane recycling (e.g., *RAB11B*). Table 1 reports the top-ranked upregulated genes; the full list is available in Supplementary Table S1.

**TABLE 1:** Differential gene expression analysis summarizing the top significantly upregulated endometrial genes in pregnant mares around day 10, including standardized gene nomenclature, unique genomic identifiers, expression changes, and statistical evidence supporting differential regulation.

Gene symbol	Gene ID	Entrez ID	logFC	AveExpr	t-statistic	P.Value	adj.P.Val	B-statistic
*ROPN1L*	ENSECAG00000011750	100146663	3.245128	5.3115994	4.601849	6.60E-06	6.64E-03	3.5847055
*PUM3*	ENSECAG00000017193	100058365	4.485933	4.2958947	5.200474	4.00E-07	1.03E-03	5.9820692
*SSUH2*	ENSECAG00000039589	100058920	3.647877	5.0032624	4.986555	1.10E-06	2.29E-03	5.1731106
*C1H1orf131*	ENSECAG00000024809	100060794	6.573371	2.4861318	4.944364	1.40E-06	2.33E-03	4.2037986
*ENSECAG00000027857*	ENSECAG00000027857	100059835	6.612526	3.5559473	6.098259	0.00E+00	1.99E-05	9.5896117
*SKAP1*	ENSECAG00000010544	100069371	2.925046	6.2663907	4.367898	1.83E-05	1.22E-02	2.6618541
*ENSECAG00000015109*	ENSECAG00000015109	ENSECAG00000015109	3.041506	7.1435017	4.31128	2.32E-05	1.37E-02	2.4469752
*SQOR*	ENSECAG00000008826	100070480	3.344454	4.8288769	4.250669	3.00E-05	1.67E-02	2.2117929
*ENSECAG00000032188*	ENSECAG00000032188	100050760	3.908603	4.6759508	4.195518	3.77E-05	1.89E-02	1.9586055
*ENSECAG00000020565*	ENSECAG00000020565	100057416	4.15608	4.3503034	4.1557	4.44E-05	2.05E-02	1.7727379
*KIF9*	ENSECAG00000025122	100064806	4.936011	3.3963162	4.071585	6.24E-05	2.41E-02	1.3154464
*RAB11B*	ENSECAG00000000716	100146180	2.945073	5.6299077	4.05397	6.70E-05	2.49E-02	1.4961481
*SMIM12*	ENSECAG00000023494	100069744	2.766266	5.4954826	3.960391	9.72E-05	3.05E-02	1.1541598
*COPS6*	ENSECAG00000008997	100068657	2.610697	6.1149531	3.926147	1.11E-04	3.19E-02	1.0251562
*ENSECAG00000034606*	ENSECAG00000034606	111772161	4.260807	5.0125283	4.853083	2.10E-06	3.05E-03	4.5127438
*HRAS*	ENSECAG00000008473	100055481	6.393452	3.0658238	4.762404	3.20E-06	4.04E-03	3.5157609
*ENSECAG00000014465*	ENSECAG00000014465	ENSECAG00000014465	5.928828	3.2303248	4.466172	1.20E-05	9.06E-03	2.5020464
*C4BPA*	ENSECAG00000013486	100056222	3.191632	5.7312545	4.497929	1.04E-05	9.06E-03	3.1702735
*U2AF1L4*	ENSECAG00000017941	100051464	3.215203	5.5115542	4.454111	1.26E-05	9.06E-03	2.9977135

The table reports the Gene Symbol, the official abbreviation assigned by the HUGO Gene Nomenclature Committee; Gene ID, the reference genome annotation identifier (e.g., Ensembl Gene ID) mapping each gene to its genomic locus; Entrez ID, the NCBI numerical identifier enabling cross-database integration; logFC, the log2 fold change in expression between experimental conditions, where positive values indicate upregulation and negative values indicate downregulation; AveExpr, the average log2 expression level across all samples; t-statistic, the test statistic from the linear modeling framework quantifying the magnitude of differential expression relative to its variability; P.Value, the raw p-value representing the probability of observing the measured effect under the null hypothesis; adj.P.Val, the multiple-testing–corrected p-value (Benjamini–Hochberg false discovery rate) controlling for false positives across the full gene set; and B-statistic, the Bayesian log-odds score reflecting the posterior probability that a gene is truly differentially expressed, with higher values indicating stronger statistical support. Only endometrial genes with a B-statistic>1 are included in this table, these are considered high-confidence differentially expressed genes, corresponding to a posterior probability greater than approximately 73% that the gene is truly differentially expressed.

#### Downregulated endometrial genes

Downregulated endometrial genes with strong statistical support (B > 1) were dominated by immediate-early and stress-responsive regulators (*EGR1, FOS, DUSP1*), detoxification/oxidative stress handling (*GSTA4*), and chromatin regulation (*KMT2C*) (Table 2; Supplementary Table S1). Notably, the concerted suppression of *EGR1, FOS,* and *DUSP1* is consistent with attenuation of acute MAPK-driven transcriptional responsiveness, suggesting a transition toward transcriptional stabilization in the pregnant endometrium during this window.

**TABLE 2:** Most significantly downregulated genes in the endometrium of pregnant mares identified by differential expression analysis.

Gene symbol	Ensembl ID	logFC	AveExpr	t-statistic	P.Value	adj.P.Val	B-statistic
GSTA4	ENSECAG00000019037	−5.91	5.81	−6.12	9.00E-06	1.99E-05	10.3
EGR1	ENSECAG00000014338	−4.71	7.38	−5.79	1.74E-05	6.94E-05	8.85
SLC12A2	ENSECAG00000012432	−4.38	6.2	−4.13	4.92E-05	2.15E-02	1.73
DUSP1	ENSECAG00000009558	−3.88	6.2	−4.13	4.92E-05	2.15E-02	1.73
KMT2C	ENSECAG00000029521	−5.13	5.87	−4.04	7.16E-05	2.57E-02	1.58
FOS	ENSECAG00000017807	−3.82	6.2	−4.13	4.92E-05	2.15E-02	1.73
MAPK4	ENSECAG00000037479	−3.82	6.2	−4.13	4.92E-05	2.15E-02	1.73

The table lists the subset of transcripts showing the strongest evidence of downregulation (logFC < –2.5; FDR-adjusted P < 0.05) in pregnant versus non-pregnant mare endometrium. Statistical significance was assessed using linear modeling with empirical Bayes moderation, providing robust variance estimation despite moderate sample sizes. The t-statistics and corresponding adjusted P-values (Benjamini–Hochberg correction) reflect high statistical power across genes with moderate to high expression levels (AveExpr), minimizing the likelihood of false positives. The B-statistic (log-odds of differential expression) further quantifies the strength of evidence, with several genes (e.g., GSTA4, EGR1) showing exceptionally high posterior probabilities of true differential regulation. Functionally, the downregulated genes encompass key pathways relevant to early pregnancy, including oxidative stress response (GSTA4), transcriptional regulation and immediate-early signaling (EGR1, FOS), ion and amino acid transport (SLC12A2), MAPK pathway modulation (DUSP1, MAPK4), and chromatin remodeling (KMT2C). Collectively, these genes represent core components of the endometrial molecular shift associated with early gestational adaptation.

### Transcriptomic dynamics of equine embryos from day 8 to 12

Transcriptomic profiling of equine embryos from Day 8 to Day 12 revealed a robust and coordinated induction of genes with established roles in endocrine competence and interface construction. High-confidence upregulated transcripts (B > 1) included *SERPINE1, TMPRSS2, CYP19A1, CYP17A1,* and *CDS2*, together with extracellular matrix and basement membrane components (Table 3) (*COL4A1, COL4A2, COL4A5, SPON1*) and adhesion-related *DSG2*. Selective transport specialization was supported by *AQP5, SLC2A5,* and *ATP1B3*, consistent with regulated water, solute, and ion handling at the embryonic surface, and protein processing and glycosylation by *B4GALT1*, *GCNT2*, *FUT8*, and *LGMN*. Collectively, these transcriptional changes reflect a marked increase in structural, metabolic, and transport capacity as embryos transition from the mobile blastocyst stage toward fixation and early attachment. Additional significantly regulated genes (FDR < 0.05) are provided in Supplementary Table S1; however, genes with B ≤ 1 were not used as primary drivers of the conceptual model. A smaller subset of transcripts was downregulated (e.g., *DNMT3B, E2F8*), consistent with shifts in transcriptional and proliferative programs as embryos progress toward fixation and early attachment (Table 3).

**TABLE 3:** Most significantly upregulated genes in equine embryos between Day 8 and Day 12*.*

Gene symbol	Ensembl ID	logFC	AveExpr	t-statistic	P.Value	adj.P.Val	B-statistic
SERPINE1	ENSECAG00000019781	6.8225391	9.05615	7.010374	6.40E-06	3.24E-03	4.1960498
TMPRSS2	ENSECAG00000022289	6.7899828	7.070928	6.352667	1.85E-05	5.29E-03	3.1544558
CYP19A1	ENSECAG00000020474	6.6950084	8.836498	10.613303	0.00E+00	2.18E-04	8.6754583
CDS2	ENSECAG00000012485	6.6307866	7.14231	8.740587	5.00E-07	7.77E-04	6.4094276
CYP17A1	ENSECAG00000010022	6.4571917	6.799185	6.168398	2.52E-05	6.77E-03	2.8727172
COL4A5	ENSECAG00000034852	6.0346962	7.625915	7.901076	1.70E-06	1.52E-03	5.415821
SPON1	ENSECAG00000020789	5.8012633	6.272299	5.642908	6.24E-05	9.26E-03	2.0155662
FMO1	ENSECAG00000006012	5.6292205	6.294656	8.73761	5.00E-07	7.77E-04	6.2556832
CTBS	ENSECAG00000015643	5.6091644	10.452344	6.865642	8.00E-06	3.67E-03	3.9831638
AQP5	ENSECAG00000008500	5.520652	6.868312	5.929593	3.79E-05	6.92E-03	2.4936371
NEU2	ENSECAG00000030943	4.6833274	6.283617	6.390506	1.74E-05	5.29E-03	3.1896637
PYGL	ENSECAG00000011728	4.6336671	6.346873	5.788966	4.83E-05	7.88E-03	2.2598732
HNF4A	ENSECAG00000024450	4.4521824	5.996885	5.051017	1.81E-04	2.07E-02	1.0278725
COL4A2	ENSECAG00000021314	4.1532988	6.888749	5.96491	3.56E-05	6.92E-03	2.5555818
COL4A1	ENSECAG00000019838	3.9633199	7.285549	5.199134	1.38E-04	1.75E-02	1.2685208
DSG2	ENSECAG00000013616	3.8844093	6.815873	7.088372	5.70E-06	3.24E-03	4.2524597
SLC2A5	ENSECAG00000022093	3.8352276	5.892059	5.646449	6.20E-05	9.26E-03	2.0188346
B4GALT1	ENSECAG00000012393	3.5449838	7.332661	6.513749	1.42E-05	5.03E-03	3.430443
PKP2	ENSECAG00000013566	3.5042833	7.413155	6.466358	1.53E-05	5.03E-03	3.346562
GREB1L	ENSECAG00000019278	3.313398	8.10169	5.63803	6.29E-05	9.26E-03	2.0130011
FUT8	ENSECAG00000014561	3.2533708	6.961511	6.123284	2.72E-05	6.90E-03	2.8109785
DNASE1L3	ENSECAG00000015857	3.1610876	7.005176	5.12574	1.58E-04	1.90E-02	1.1497905
PUDP	ENSECAG00000009014	3.1179536	9.11004	5.19993	1.38E-04	1.75E-02	1.2298863
TCN2	ENSECAG00000020874	3.1054563	10.149954	6.080542	2.93E-05	6.92E-03	2.7312717
LGMN	ENSECAG00000010227	2.9967671	8.987737	5.082391	1.71E-04	2.00E-02	1.0191629
CKAP4	ENSECAG00000006881	2.9893866	9.248406	5.154404	1.50E-04	1.85E-02	1.147054

The table summarizes the transcripts showing the strongest upregulation during early equine embryonic development, ranked by statistical significance and magnitude of fold change. All genes listed passed an FDR threshold of <0.05 and exhibited high statistical power, as reflected by large t-statistics and positive B-statistics (log-odds of true differential expression). Average expression values (AveExpr) indicate robust transcript abundance across replicates, minimizing the likelihood of low-expression artefacts.

### Enrichment analysis

**FIGURE 1:**
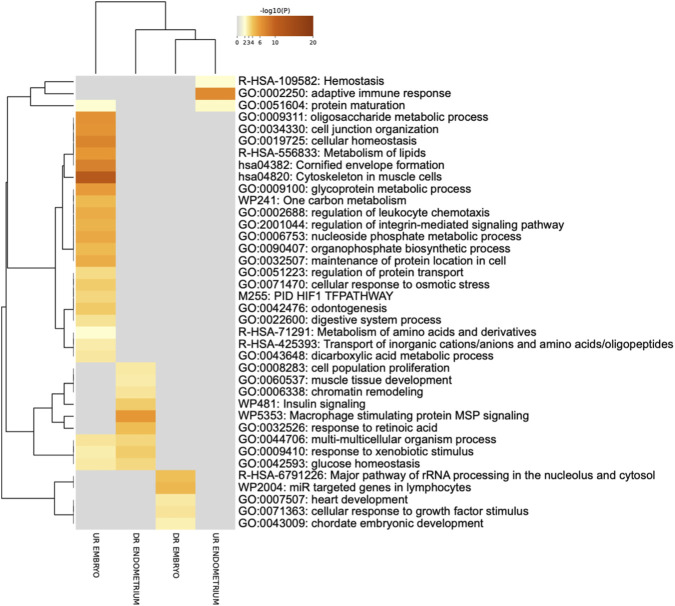
Functional enrichment heat map of upregulated (UR) and downregulated (DR) genes in embryo and endometrium. The heat map displays the top enriched Gene Ontology, Reactome, KEGG, and WikiPathways terms identified by Metascape for UR and DR gene sets in the embryo and endometrium. Warmer colors indicate stronger enrichment (higher–log10(P)). UR embryo genes activate developmental, biosynthetic, and chromatin-remodeling pathways that enhance embryonic signaling capacity, while DR embryo genes suppress stress-response and environmental-sensing functions. UR endometrium genes highlight immune modulation, integrin-mediated adhesion, hemostasis and protein maturation, reflecting maternal responses to embryo-derived cues. DR endometrium genes show reduced metabolic and transport activity, consistent with resource reallocation toward receptivity. Together, these directional enrichments illustrate a reciprocal embryo–maternal molecular dialogue essential for peri-implantation success.

To maximize functional interpretability, enrichment analysis was performed using human orthologs, leveraging the greater depth of annotation in *Homo sapiens*. Orthology mapping is described in Materials and Methods. Gene set enrichment was performed with Metascape, and we report the top-ranked terms for upregulated and downregulated gene sets in both embryo and endometrium. Enrichment results are summarized in Figure 1.

## Discussion

This study integrates matched RNA-seq profiles of early pregnancy progression (days 8–12 in the embryo) and endometrium transcriptome to refine the timing and molecular features of early embryo–maternal communication in the mare. Rather than revisiting whether cross-talk exists, which is supported by classical physiology and prior transcriptomic studies (Aurich and Budik, 2015; Klein, 2015; 2016; Conley and Ball, 2019; Swegen, 2021), we aimed to identify when the interaction becomes transcriptionally detectable and which high-confidence gene programs (B > 1) are engaged.

**FIGURE 2:**
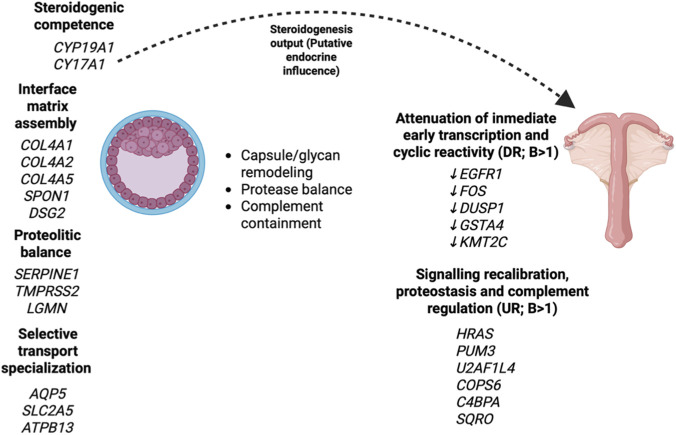
Coordinated bilateral reprogramming of conceptus and endometrium during maternal recognition of pregnancy in the mare. Schematic representation integrating statistically robust differentially expressed genes (B > 1) from matched conceptus (Days 8–12) and endometrial transcriptomes during the previous to the maternal recognition of pregnancy. Left panel (Conceptus): The conceptus exhibits acquisition of endocrine competence, evidenced by induction of steroidogenic enzymes CYP19A1 and CYP17A1, together with coordinated assembly of extracellular matrix and interface components (COL4A1, COL4A2, COL4A5, SPON1, DSG2). The regulation of proteolytic balance is indicated by the strong upregulation of SERPINE1, TMPRSS2, and LGMN, consistent with the restraint of invasive pathways. Selective transport specialization is supported by increased expression of AQP5, SLC2A5, and ATP1B3, reflecting regulated water, solute, and ion handling at the embryonic surface. Collectively, these changes indicate active endocrine output and remodeling of the structural interface by the conceptus. The embryo–maternal boundary is conceptualized as a dynamic interface characterized by capsule/glycan remodeling, extracellular matrix organization, protease balance, and complement containment. Arrows indicate putative conceptus-derived influences on the maternal environment without implying direct causal hierarchy. Right panel (Endometrium): The maternal transcriptome is defined by attenuation of immediate-early and stress-responsive transcriptional programs, including downregulation of EGR1, FOS, DUSP1, GSTA4, and KMT2C, consistent with reduced acute MAPK-driven responsiveness and transcriptional stabilization. Concurrently, selective upregulation of signaling and regulatory components (HRAS, PUM3, U2AF1L4, COPS6), complement modulation (C4BPA), and mitochondrial sulfide metabolism (SQOR) suggests controlled remodeling, recalibration of intracellular signaling, and maintenance of immune containment rather than inflammatory activation. Together, the model illustrates coordinated but asymmetric adaptation during early equine pregnancy, in which conceptus endocrine and interface specialization is coupled to maternal transcriptional stabilization and regulated tissue remodeling. The figure reflects only transcripts meeting a Bayesian posterior probability threshold of B > 1, ensuring high-confidence differential expression.

Biologically, these data suggest that the onset of a single dominant embryo–maternal transcriptional event does not define maternal communication in the mare, but rather by coordinated and compartment-specific changes occurring before the classical prostaglandin-dependent phase of maternal recognition. The induction of conceptus genes involved in steroidogenesis, extracellular matrix organization, protease regulation, and selective transport indicates progressive acquisition of endocrine and interface competence. In parallel, the pregnant endometrium showed attenuation of immediate-early transcriptional programs and selective upregulation of genes involved in signaling, complement regulation, and redox-linked metabolism. This pattern is consistent with controlled maternal recalibration rather than overt inflammatory activation. Therefore, the main contribution of this study is to provide a matched, systems-level framework showing that conceptus maturation and maternal adaptation are temporally coordinated but biologically asymmetric during the onset of equine pregnancy (Figure 2).

The mare provides a distinctive reproductive context in which the conceptus remains mobile within the uterine lumen until fixation, and no single molecular mediator of maternal recognition of pregnancy has been established. This biology is compatible with a distributed, multi-factorial communication strategy rather than a single linear endocrine signal. Within this framework, the present findings support a temporally progressive model in which conceptus endocrine and interface specialization develops in parallel with maternal transcriptional stabilization and controlled tissue remodeling.

The endometrial response is characterized by strong downregulation of immediate-early transcription factors and MAPK feedback control (*EGR1, FOS, DUSP1*), together with reduced expression of oxidative stress handling (*GSTA4)* and chromatin regulation (*KMT2C*). In parallel, upregulated endometrial transcripts include complement regulation (*C4BPA*), signaling and regulatory nodes (*HRAS, PUM3, U2AF1L4, COPS6*)*,* and redox-linked metabolism (*SQOR).* Collectively, this pattern is most consistent with attenuation of acute transcriptional responsiveness and controlled remodeling rather than overt inflammatory activation, broadly aligning with the restrained uterine response described in microarray studies of early equine pregnancy (Klein et al., 2010; Merkl et al., 2010). The coordinated suppression of *EGR1–FOS* together with *DUSP1* suggests reduced MAPK-driven immediate-early signaling, potentially reflecting stabilization of the luminal epithelial environment during conceptus mobility.

Concurrently, the conceptus exhibits robust induction of steroidogenic enzymes (*CYP19A1, CYP17A1),* consistent with increasing steroidogenic capacity during the peri-recognition interval, as previously reported for equine conceptuses (Merkl et al., 2010; Klein and Troedsson, 2011). Although a direct role of conceptus-derived steroids in equine MRP remains unproven, enhanced steroidogenic potential may contribute to local modulation of the uterine milieu during this transitional phase. Conceptus genes with the strongest statistical support also indicate active construction of the embryo–maternal interface through basement membrane and extracellular matrix assembly (*COL4A1, COL4A2, COL4A5, SPON1, DSG2*), together with glycan remodeling (*FUT8, B4GALT1*). The prominent induction of *SERPINE1,* alongside *TMPRSS2* and *LGMN*, supports tight regulation of extracellular proteolytic balance, a theme biologically consistent with the prolonged, non-invasive pre-attachment period in the horse.

Several conceptus transcripts highlighted here (e.g., *CYP19A1, AQP5, SLC2A5, COL4A1/2, SPON1, FUT8*, and the capsule-linked enzyme *NEU2*) were also reported as upregulated in the conceptus microarray study of Klein and Troedsson (2011)(Klein and Troedsson, 2011), supporting cross-platform reproducibility and reinforcing the concept of an emerging extracellular/secretory phenotype during early pregnancy. The upregulation of *FUT8* and *NEU2* further suggests that glycan remodeling may be an important component of conceptus maturation during this period. *FUT8* encodes an α1,6-fucosyltransferase involved in core fucosylation of N-linked glycans, whereas *NEU2* encodes a sialidase that can modulate the sialylation state of glycoconjugates. Although the present transcriptomic data do not directly define glycan composition or enzymatic activity, these changes are consistent with remodeling of glycoproteins and glycoconjugates at the conceptus surface. Such remodeling may influence extracellular matrix organization, capsule-associated properties, ligand–receptor interactions, and immune recognition at the embryo–maternal interface. Therefore, *FUT8* and *NEU2* represent potentially relevant candidates for future studies focused on conceptus glycobiology during early equine pregnancy.

Comparison with the endometrial microarray study of Klein and Troedsson (Klein and Troedsson, 2011) highlights both convergence and divergence. Klein and Troedsson. Emphasized induction of transport and secretory programs (including *SLC36A2* and *IGFBP1*) and reduced ESR1 expression at Day 13.5, whereas our Day-8 to 12-RNA-seq data prioritize attenuation of immediate-early responsiveness (*EGR1/FOS/DUSP1)* together with selective upregulation of complement regulation and intracellular signaling/trafficking nodes. These differences may reflect stage, sampling region, and platform effects, but together support the view that the equine uterine response to pregnancy is dynamic and multi-dimensional rather than reducible to a single pathway.

Notably, we did not detect high-confidence differential expression of core prostaglandin synthetic enzymes or oxytocin receptor transcripts at this early stage. Given that functional suppression of endometrial PGF_2_α secretion characterizes the classical maternal recognition window in the mare, this observation suggests that decisive prostaglandin regulatory events may occur slightly later in gestation or be mediated predominantly through post-transcriptional and signaling-level mechanisms rather than large transcriptomic shifts. This interpretation is consistent with a distributed and temporally progressive model of equine maternal recognition rather than a single discrete transcriptional switch.

Taken together, our matched datasets support coordinated but asymmetric adaptation at the onset of early pregnancy: the conceptus increases endocrine and interface competence while the endometrium transitions toward transcriptional stabilization with controlled immune containment and remodeling. The limited direct gene overlap between compartments is expected; the strength of the matched sampling design lies in identifying convergent functional themes—proteolytic balance, interface remodeling, immune containment, and signaling recalibration—that can be prioritized for targeted validation.

Recent transcriptomic and proteomic datasets of equine endometrium and conceptus across days 8–14 likewise report moderate gene-level overlap across cohorts but recurring pathway-level themes involving metabolism, immune modulation, and tissue remodeling (Gibson et al., 2020; Vegas et al., 2022; de la Fuente et al., 2023; Ulaangerel et al., 2025). Our results extend this literature by anchoring conceptus and maternal signatures within the same biological window and applying a stringent posterior probability filter, thereby providing a conservative, high-confidence set of candidate pathways for mechanistic follow-up. Overall, these findings are compatible with a multi-factorial and temporally progressive model of equine maternal recognition in which endocrine output, interface assembly, and immune containment emerge in parallel before the classical prostaglandin suppression phase.

## Data Availability

The datasets presented in this study can be found in online repositories. The names of the repository/repositories and accession number(s) can be found in the article/Supplementary Material.
